# Aging and Sensory Substitution in a Virtual Navigation Task

**DOI:** 10.1371/journal.pone.0151593

**Published:** 2016-03-23

**Authors:** S. Levy-Tzedek, S. Maidenbaum, A. Amedi, J. Lackner

**Affiliations:** 1 Recanati School for Community Health Professions, Department of Physical Therapy, Ben Gurion University of the Negev, Beer-Sheva, Israel; 2 Zlotowski Center for Neuroscience, Ben Gurion University of the Negev, Beer-Sheva, Israel; 3 Department of Medical Neurobiology, Institute for Medical Research Israel-Canada, Faculty of Medicine, Hebrew University of Jerusalem, Jerusalem, Israel; 4 Department of Cognitive Science, Faculty of Humanities, Hebrew University of Jerusalem, Jerusalem, Israel; 5 Sorbonne Universités UPMC Univ Paris 06, Institut de la Vision, Paris, France; 6 Ashton Graybiel Spatial Orientation Laboratory, Department of Physiology, Brandeis University, Waltham, Massachusetts, United States of America; University of Bath, UNITED KINGDOM

## Abstract

Virtual environments are becoming ubiquitous, and used in a variety of contexts–from entertainment to training and rehabilitation. Recently, technology for making them more accessible to blind or visually impaired users has been developed, by using sound to represent visual information. The ability of older individuals to interpret these cues has not yet been studied. In this experiment, we studied the effects of age and sensory modality (visual or auditory) on navigation through a virtual maze. We added a layer of complexity by conducting the experiment in a rotating room, in order to test the effect of the spatial bias induced by the rotation on performance. Results from 29 participants showed that with the auditory cues, it took participants a longer time to complete the mazes, they took a longer path length through the maze, they paused more, and had more collisions with the walls, compared to navigation with the visual cues. The older group took a longer time to complete the mazes, they paused more, and had more collisions with the walls, compared to the younger group. There was no effect of room rotation on the performance, nor were there any significant interactions among age, feedback modality and room rotation. We conclude that there is a decline in performance with age, and that while navigation with auditory cues is possible even at an old age, it presents more challenges than visual navigation.

## Introduction

Sensory substitution devices (SSDs) convey information that is usually perceived by one sense, using an alternative sense [1]. For example, auditory cues can be used to convey information that is usually perceived using visual cues. In recent years, there has been a surge of studies on the use of sensory substitution devices (e.g., [2–4]), but none of them, to the best of our knowledge, examined the effects of aging on the ability to use SSDs, or have challenged the use of SSDs by introducing a spatial perception bias while performing a task with the device.

In the current study we made a first step towards bridging this gap. We studied the differences between younger and older adults who perform a navigation task in a virtual environment using sensory substitution. We further explored whether use of SSDs is susceptible to the introduction of external interference in the form of a spatial perception bias.

We thus connected previously parallel lines of research, on sensory feedback, aging, virtual navigation and spatial cognition. Specifically, we asked what are the combined effects of age, sensory modality and spatial-perception bias on movement through a virtual maze (our specific hypotheses are detailed below). We addressed these questions in a single experiment, rather than a series of separate experiments, so that we can examine not only main effects of each factor (age, sensory modality and spatial-perception bias), but also the interaction effects between these factors.

### Aging and movement

Aging is accompanied by a gradual decline in selective cognitive functions, including attention, information processing, and learning [5], while functions such as semantic memory, comprehension and vocabulary can remain stable or may even improve with age [6–7].There are changes in the quality of movement control that take place with age. For example, movements are slower (e.g., [8]), there is evidence of reduced movement planning, and more reliance on visual feedback during movement execution [9]. Complex tasks (e.g., navigation), which engage several aspects of cognition and motor planning, are consequently affected. At least some of these changes in task performance–and particularly in navigational abilities–might be explained by degradation of brain structures involved in navigation; particularly, the hippocampus, the parahippocampal gyrus, the posterior cingulate gyrus, the parietal lobes and the pre-frontal cortex [10]. When navigating through space, we must maintain a representation of our position with relation to the three-dimensional world, a task that draws on our spatial cognition abilities. We tap into these abilities whenever we navigate to a familiar place, or learn the route to a new one. We therefore rely on our spatial cognitive abilities in our everyday life ubiquitously. We regularly encounter novel environments, and the process of adjusting our movement plan for navigating within them based on their layout, location of obstacles, and moving entities, is highly dynamic. And yet, testing of cognitive aging has focused on static, paper-based tests, rather than dynamic ones [10]. In order to evaluate spatial cognition, a dynamic test is better suited to uncover the ability to efficiently interpret dynamically changing spatial information.

### Virtual navigation

Virtual environments have been used in recent years as a tool to study navigation patterns in simple and complex settings. Several researchers have put particular emphasis on making them accessible to blind and visually impaired individuals–via auditory and tactile cues–so they can be used to study negotiation of space in the absence of visual cues [11–17]. In fact, virtual mazes can, in and of themselves, be used as a rehabilitation tool, for at-home training to navigate through an unfamiliar environment, for individuals with visual impairment (e.g., [11]). In this scenario, visual information is conveyed via sounds, in what is termed "sensory substitution" (e.g., [13–14, 18–19]). It has been demonstrated that learning of new environments in the virtual realm transfers to the corresponding real-world setting [11, 15, 20].

This simple yet powerful tool can be used to study perception of spatial cues arriving from different sensory modalities.

### Rotation and biased perception of space

The Slow Rotation Room at Brandeis University has been developed as a tool to study human orientation, movement control, and perception during exposure to angular and linear acceleration.

When exposed to angular acceleration in a dark environment, people experience the oculogyral illusion. In this situation, a head-fixed visual target will appear to move through space and be displaced relative to the person’s body in the direction of acceleration [21]. However, an audio signal that is presented at a person’s midline position during acceleration, will be perceived as if it has been displaced in the direction *opposite* to the direction of acceleration; this phenomenon is known as the audiogyral illusion [21]. Body localization is also affected under these circumstances, and this effect is referred to as the somatogravic illusion. Lackner & Dizio [22] have shown that the three illusions are correlated in extent and direction, suggesting that there is an internal remapping of a common reference frame for visual, auditory, and haptic localization in the accelerated environment. In other words, a bias in spatial perception is generated, across the senses, as a result of exposure to acceleration. We set out to test the effect of this perceptual bias on the performance of a motor task. Specifically, we tested the effect of the perceptual bias on navigation within a virtual maze when spatial information is provided either visually or auditorily.

To the best of our knowledge, this is the first experiment to study effects of age on using a sensory-substitution device, and the first to use a dynamically changing virtual environment within a rotating room setting.

### Hypotheses

Our hypotheses were that:

1. Older adults will have a reduced ability to interpret auditory cues representing visual information, compared to young adults. Thus, we expect younger adults will perform better performance on the virtual-maze task than older adults.
2. Auditory cues will be more difficult to interpret as indicators of distance than visual information. Thus, we expect performance on the virtual-maze task will be better when visual cues are available, than when auditory cues are available.
3. Introducing a spatial perception bias (via centripetal force) will interfere with the performance on a motor task. Thus, we expect performance on the virtual maze game when the room is rotating to be worse than the performance on the task when the room is stationary.

Our outcome measures for these hypotheses were a series of indicators of success on a virtual navigation task, such as the time it took to complete the virtual maze, and the number of collisions with the maze walls. The specific performance metrics are detailed below.

## Materials and Methods

### Equipment

#### The eye cane

Sensory feedback to the participants, as they were navigating through the virtual mazes (described below), was given either using visual cues or auditory cues. Auditory cues were provided using the "virtual EyeCane" [14]. The virtual EyeCane is based on a physical sensory-substitution device, called the EyeCane [12]. The physical EyeCane is a hand-held device, which uses infra-red sensors to detect distance from objects located up to 5 meters away from the user. The device reports the distance from the object at which it is pointed by producing a series of beeps; the closer an object is to the user, the higher is the frequency of the beeps. For example, if users were to stand 5 meters from a wall while pointing the device at it, then gradually approaching it, they would initially not receive any auditory feedback (outside the range of the device), and as they would get closer to the wall, the device would start beeping, with the beeps sounded out closer together the closer the users get to the wall.

The virtual EyeCane is a virtual representation of the physical EyeCane. It produces a series of beeping sounds at a frequency which is determined by the distance of the virtual device from the virtual object at which it is pointed. The participants were represented by 'avatars' as they navigated through virtual mazes. The avatars held the virtual EyeCane in their hands (the device was not visible to the participants), and the device was always pointed at the direction at which the avatar was facing. The avatar was free to rotate right and left while walking through the mazes.

The virtual EyeCane conveys distances up to 5 virtual meters to the user by changing the frequency of a series of beeping sounds, such that 5 virtual meters and up will be silent, and the closer the object the device points at, the higher is the beeping rate.

#### The virtual mazes

The virtual mazes were created using Blender 2.49 and Python 2.6.2. The location and orientation of the participants' avatar was tracked and recorded at 20 Hz.

The avatar representing the participants was not visible, and they had first-person view of the mazes. That is, the visual input was similar to what they would see if they actually navigated through a maze in the real world. Navigation was accomplished using the arrow keys on a standard laptop keyboard, and the auditory cues were delivered via standard headphones. The participants heard a collision sound whenever their avatar came in contact with a virtual wall. Distances within the environment were set so that each 'virtual meter' corresponds to a real-world meter. Thus, the auditory output from the Virtual-EyeCane at one 'virtual meter' is the same as the output from the real-world EyeCane at a distance of one meter.

The participants controlled their location within the maze using the keyboard in the following way: The 'up' and 'down' arrow keys advanced the avatar forward and backward, respectively, and the 'left' and 'right' arrow keys rotated the avatar in the respective direction. The participants could control the speed of their navigation through the mazes by controlling the speed at which they clicked on the arrow keys.

A video demonstrating navigation through a visual and an auditory maze can be found in S1 Video.

#### The rotating room

The experiment was conducted in the Brandeis Rotation Room, a fully enclosed circular room, 6.7 m in diameter. In this room, when participants are rotated at a constant velocity in a fully enclosed environment, they feel as if they are stationary in a stationary environment, but feel heavier than normal and experience some body tilt. For each participant, half of the trials were conducted with the room being stationary, and half with the room rotating about its central axis. For the rotation trials, the room was accelerated at 1°/s^2^ to a velocity of 60°/s (10 rpm), and held there for 1 minute before starting the rotation block of trials. Upon completion of the rotation block of trials, the room was decelerated at 1°/s^2^ to a stop. The rotation was in the counterclockwise direction. Participants were explicitly aware of the rotation of the room during the rotation trials.

### Participants

Twenty nine participants took part in the experiment. Seventeen young adults (19–23 years old, mean±SD: 20.7±1.3 years; 11 males, 6 females) and 12 older adults (58–69 years old, 64.3±3.7 years; 8 males, 4 females). Four of the senior adults and one of the younger adults have previously been in the rotating room. Individuals who suffered from high blood pressure, claustrophobia, respiratory problems, ADHD, had a history of seizures or problems with balance were excluded from participation. All participants gave their written informed consent to participate.

### Procedure

Participants were screened for medical conditions that would preclude their participation according to the exclusion criteria listed above. Their blood pressure was collected using a commercially available automated blood pressure cuff.

**Fig 1:**
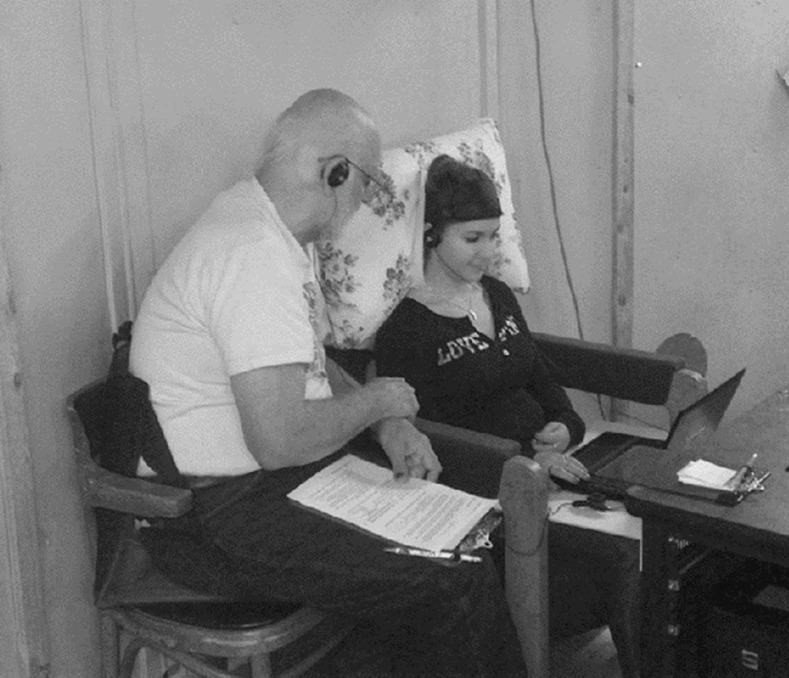
Experimental setup. The participant is seated in the periphery of the Rotating Room, with comfortable head support. She is controlling the movement of the avatar through a virtual maze using the arrow keys of a laptop placed on a flat support located on her lap.

Participants were seated comfortably on a chair, padded with pillows for the back and the head, with a laptop computer placed on their lap, on top of a rigid support (see Fig 1). The chair was located at the periphery of the rotating room. The entire experimental procedure took place in a single session inside the rotating room. In all but one case, two participants were tested in parallel, on opposite sides of the rotating room. Each participant was accompanied by an experimenter for the entire duration of the experiment. The experimental protocol was approved by the Internal Review Board (IRB) of the Brandeis University.

#### Training session

The algorithm of the virtual EyeCane was explained to the participants, and they were given the opportunity to navigate within two training mazes as many times as they wished until they felt comfortable with the task. There was no overlap between the shapes of the training mazes and shapes of the mazes used during the test session. The training phase was the only part of the experiment when participants concurrently got both visual information, presented on the computer screen, and auditory information via the headphones, using the virtual EyeCane. They were encouraged to pay attention to the correspondence between the visual and the auditory feedback, so that they could use it during the trials in which only auditory feedback will be given. They were also encouraged to experiment with closing and opening their eyes as they navigated through the maze, so they could compare their perception with their eyes closed to that with their eyes open. All training was done with the room being stationary.

#### Test session

**Fig 2:**
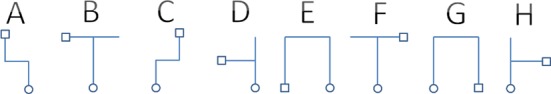
The virtual mazes. A sketch of the eight virtual mazes that participants were asked to traverse (marked as mazes A-H). A circle (◦) denotes the starting point of the maze, and a square (□) denoted the end point (exit).

Each participant was presented with eight different mazes (see Fig 2), each repeated five times consecutively, for a total of 40 experimental trials per participant. The goal was to locate the exit from the maze and virtually walk through it. The maximum allowed time for completion of a trial was 3.5 minutes, after which the trial was terminated, and the next trial was presented. After each trial, the participants were asked to draw the shape of the maze on a piece of paper, and mark the start and the end point of the maze.

**Fig 3:**
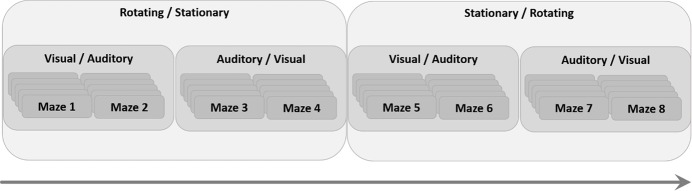
The protocol. A schematic representation of the experimental protocol. Participants started with either a “stationary room” or “rotating room” block of trials, within which they performed the first half of the trials. The block was further divided into auditory and visual blocks, within each one, the participants performed five consecutive repetitions of each of two mazes.

The experimental session was comprised of two blocks of trials: a stationary block and a rotating block. Each block comprised four mazes, two of the four mazes were presented with visual feedback ('visual condition'), and two mazes were presented with auditory feedback ('auditory condition'; See Fig 3). Each maze was repeated five times for a total of 20 trials per block. Approximately half of the participants completed the rotating block first, and half completed the stationary block first. During the auditory condition, participants wore a blindfold, and the auditory feedback was provided via headphones. A headphone-jack splitter was used, with two sets of headphones connected to it, such that one set was used by the participant, and the other by the experimenter.

The order of the mazes and the blocks was counter-balanced across participants.

### Performance metrics

The number of trials that were terminated for exceeding the maximum time limit allotted per trial (3.5 minutes) was calculated per participant. These trials were excluded from further analysis which was done using the performance metrics detailed below.

#### Time

The time to successful completion of the trial was calculated as the time elapsed between the start of movement within the maze, and until the user-controlled avatar reached the end of the maze.

#### Path length

Path length was calculated as the total distance traversed by the user’s avatar from the start to the end of the maze.

#### Number of pauses

A pause was defined as the absence of movement in the x-y plane for longer than 2 seconds. The number of pauses per trial was calculated.

#### Collisions

A collision was defined as a contact between the user’s avatar and one of the virtual walls of the maze. The number of collisions per trial was calculated.

### Statistical analysis

A Cross-Nested Mix Model GLM analysis was performed using IBM SPSS Statistics (Version 23.0. Armonk, NY: IBM Corp). The fixed-effect factors that were used in the model were the age, the feedback modality and the room rotation, with two levels for each factor. The two-way and three-way interactions among these factors were tested. The random-effect factors were the individual participants, and within the individual participants, the maze types and the repetitions within each maze. The REML method was employed for the analysis. In a case where the random effects were found not to contribute to the model, they were removed, and the model was re-fitted. To comply with the basic assumptions for the analysis, the appropriate transformation was performed for each of the performance metrics. The appropriate transformation was selected based on a Box & Cox procedure [23]. The data on the time it took to complete the mazes underwent a $\frac{1}{x}$ transformation; the data on the path length and the number of collisions underwent a $\frac{1}{{\left(x+1\right)}^{2}}$ transformation; and the data on the number of pauses underwent a $\frac{1}{{\left(x+1\right)}^{2}}$ transformation for values up to four, and a $\frac{1}{{\left(5+1\right)}^{2}}$ transformation for values greater than 4. Effect sizes in terms of *η*^2^ are not reported since they cannot be directly calculated using this statistical model in a standard manner. P values < 0.05 were considered to indicate a significant difference.

## Results

One participant in the younger group and one in the older group did not complete all trials, due to discomfort. The total number of recorded trials was therefore 1123. Of these trials, a total of 120 trials were terminated, because the participants exceeded the 3.5-min time limit allotted for completing each maze. The majority of incomplete trials was found in the auditory, rather than in the visual condition (53 trials in the auditory condition, vs. 2 in the visual condition for the younger group, and 61 vs. 4 for the older group). These incomplete trials were excluded from further analysis, and a total of 1013 trials were analyzed.

### Main effects

Both age and the feedback modality used for navigation (vision or audition) had a significant effect on all performance metrics: time, path length, number of pauses, and number of collisions. Room rotation had no significant effect on any of the performance metrics. The average values for each of the performance metrics are given in Table 1, by condition. For each value of the independent variables (age, feedback modality and rotation), data were averaged over the other independent variables. For example, the data reported for the time it took to complete the maze under the visual condition comprise all visual trials, performed by both age groups and in both rotation conditions.

**Table 1:** Average values (mean±SD) per performance metric.

Performance metric	Visual	Auditory	Young	Old	Rotating	Stationary
**Time (sec)**	37.2 ± 20.8	84.7 ± 53.3	58.1 ± 47.5	68.3 ± 46.7	59.4 ± 44.2	64.5 ± 50.3
**Path length (virtual meters)**	18.8 ± 7.8	26.2 ± 13.8	22.8 ± 13.0	22.5 ± 9.9	22.3 ± 12.5	23.0 ± 11.3
**Number of pauses**	0.1 ± 0.4	1.0 ± 1.6	0.4 ± 1.1	0.8 ± 1.5	0.5 ± 1.2	0.6 ± 1.3
**Number of collisions**	0.3 ± 0.9	1.3 ± 2.1	0.7 ± 1.8	1.0 ± 1.4	0.8 ± 1.5	0.9 ± 1.8

P-values for the main effects are detailed, per performance metric, in Table 2.

**Table 2:** P-values for the main effects per performance metric. P-values < 0.05 are marked in bold.

Performance metric	Visual vs. Auditory	Young vs. Old	Rotating vs. Stationary
**Time**	**<0.001**	**0.005**	0.51
**Path length**	**<0.001**	**0.025**	0.36
**Number of pauses**	**<0.001**	**0.004**	0.32
**Number of collisions**	**<0.001**	**<0.001**	0.95

#### Time

**Fig 4:**
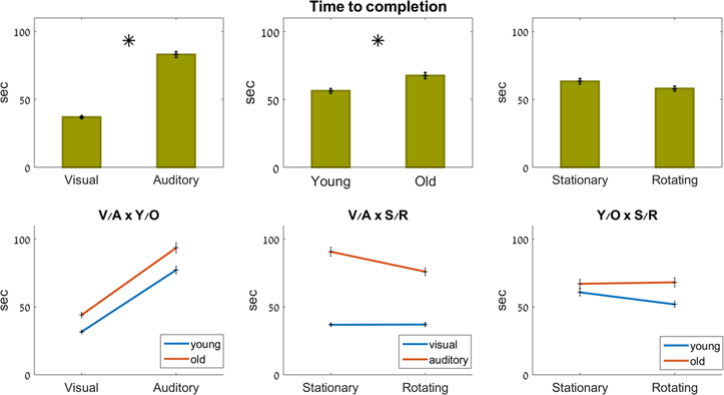
Time to completion of the mazes. Top row: main effects; bottom row: interaction effects (ns). Significant effects marked with an asterisk.

Age (F_1,27_ = 9.6, p = 0.005) and feedback modality (F_1,86_ = 170, p<0.001) both had a significant effect on the time it took to complete the mazes (see Fig 4). There was no significant effect of rotation, or of any of the interactions (p>0.4). While no significant interactions effects were present, the data from the various combinations of the independent variables are shown in graph form, for the sake of completeness.

As seen in Fig 4, bottom center pane, maze completion times were about the same for the visual trials (∼37 sec), whether performed with the room stationary or rotating. In the auditory condition, which on average took ∼85 sec to complete, maze completion times were, on average, shorter when the room was rotating, compared to when it was stationary (not significant, ns).

#### Path length

**Fig 5:**
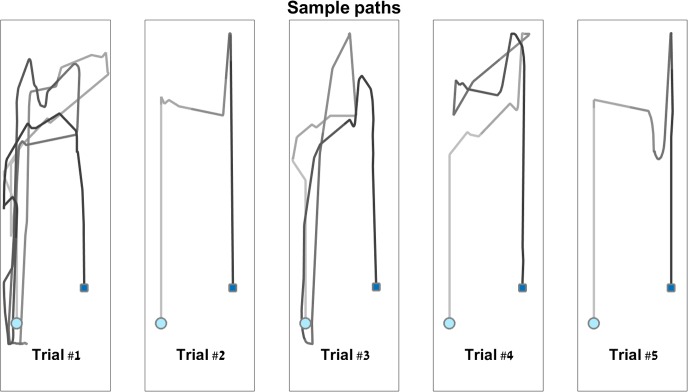
An example of actual paths traversed by the virtual avatar. Shown here are sample paths from a senior participant, completing maze G in the visual, stationary condition. Showing trials 1–5 for this maze, from left to right. A light blue circle denotes the starting point, and a dark blue square denotes the end point.

An example of the path taken by a participant over the five repetitions of a single maze is shown in Fig 5.

**Fig 6:**
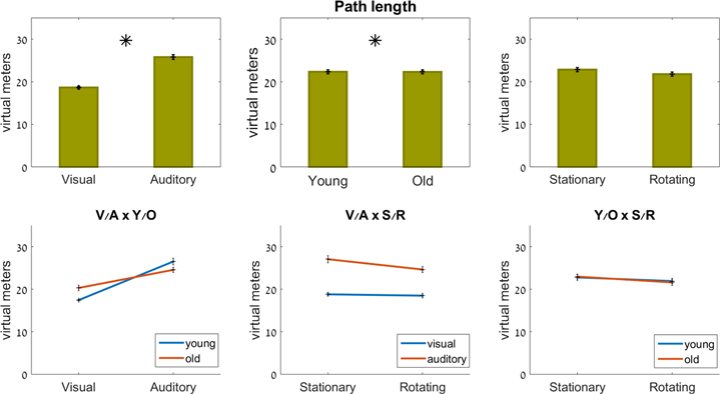
Path length. Top row: main effects; bottom row: interaction effects (ns). Significant effects marked with an asterisk.

Age (F_1,48_ = 5.4, p = 0.025) and feedback modality (F_1,244_ = 42.3, p<0.001) both had a significant effect on the path length within the virtual mazes (see Fig 6). There was no significant effect of rotation or of any of the interactions (p>0.25).

The young group moved their avatars over 18.5±5.6 virtual meters in the visually guided trials, and over 39.4±17.6 virtual meters in the trials guided by auditory feedback. The older group walked their avatars over 22.0±11.2 virtual meters in the visually guided trials, and over 36.6±10.4 virtual meters in the trials guided by auditory feedback.

As seen in Fig 6, the younger group was affected to a greater extent by the replacement of visual feedback with auditory feedback. They experienced a 57% increase in path length in the auditory vs. the visual condition, whereas the older group experienced an 18% increase in path length in the auditory condition, compared to the visual condition (ns).

#### Number of pauses

Age (F_1,26_ = 10, p = 0.004) and feedback modality (F_1,81_ = 124.3, p<0.001) both had a significant effect on the number of pauses within the virtual mazes. There was no significant effect of rotation (F_1,74_ = 1.0, p = 0.32) or of any of the interactions (p>0.07).

The young group paused, on average, 0.02±0.1 times during the visual trials, and 0.8±1.4 times during the auditory trials. The older group paused, on average, 0.2±0.5 times during the visual trials, and 1.5±1.8 times during the auditory trials. That is, the older group showed a greater increase in the number of pauses in the auditory vs. the visual condition, compared to the young group (ns).

**Fig 7:**
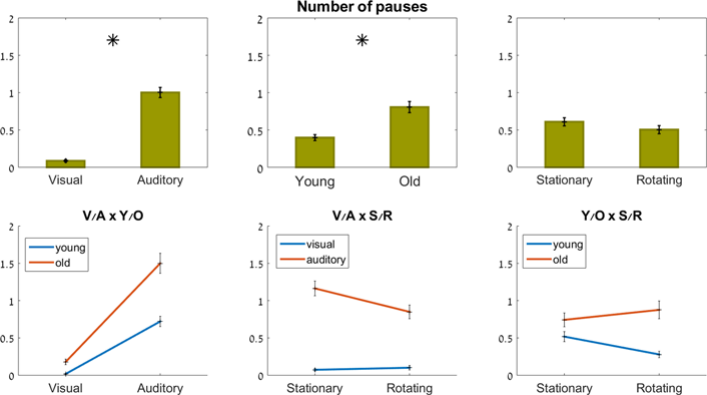
Number of pauses. Top row: main effects; bottom row: interaction effects (ns). Significant effects marked with an asterisk.

The average number of pauses per maze for the young group was 0.6±1.3 when the room was stationary, and 0.3±0.8 when the room was rotating. The older group paused on average 0.8±1.3 per maze when the room was stationary, and 0.9±1.6 per maze when the room was rotating (see Fig 7). That is, the younger group paused less when the room was rotating, whereas the older group paused less when the room was stationary (ns).

Across all participants, the number of pauses in the visual stationary condition was 0.1 ± 0.3, whereas in the visual rotating condition it was 0.1 ± 0.4. In the auditory stationary condition, participants paused 1.2 ± 1.7 times on average per maze, and in the auditory rotating condition, they paused 0.9 ± 1.5 times per maze. That is, whereas rotation did not affect the number of pauses in the visual condition, it had an unexpected effect in the auditory condition: on average, participants paused less when the room was rotating than when the room was stationary, especially the young participants (ns).

#### Number of collisions

Age (F_1,27_ = 20, p<0.001) and feedback modality (F_1,74_ = 91.1, p<0.001) both had a significant effect on the number of collisions with the virtual maze walls. There was no significant effect of rotation (F_1,71_ = 0.004, p = 0.95), or of any of the interactions (p≥0.2).

**Fig 8:**
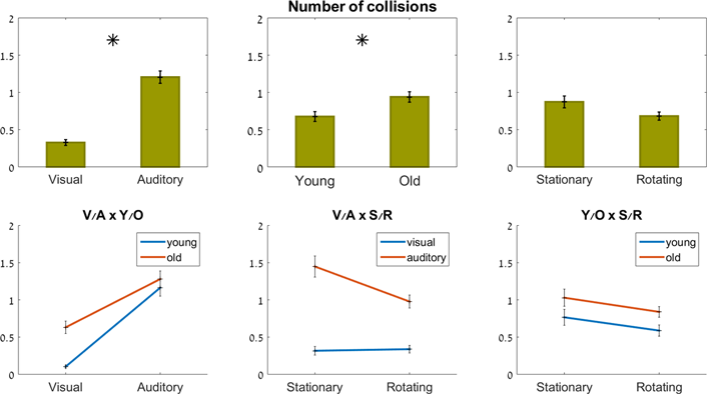
Number of collisions. Top row: main effects; bottom row: interaction effects (ns). Significant effects marked with an asterisk.

Both age groups had more collisions with the virtual walls in the auditory, compared to the visual, condition. As seen in Fig 8, bottom left, the gap between the two groups, which existed in the visual condition (the older adults making 7 times more collisions with the walls than the younger group), closed in the auditory condition, where both groups had about 1.3 collisions per maze, on average.

As shown in Fig 8, bottom middle, the rotation of the room had little effect on the number of collisions in the visual condition (∼0.35 in both stationary and rotating conditions). However, in the auditory condition, which overall had more collisions than the visual condition, there were 27% *less* collisions when the room was rotating, compared to when it was stationary (1.1±1.8 vs. 1.5± 2.3, respectively, ns).

### Path drawing

The paths drawn by participants following each trial were analyzed. No statistical analysis of the drawing data was performed.

**Fig 9:**
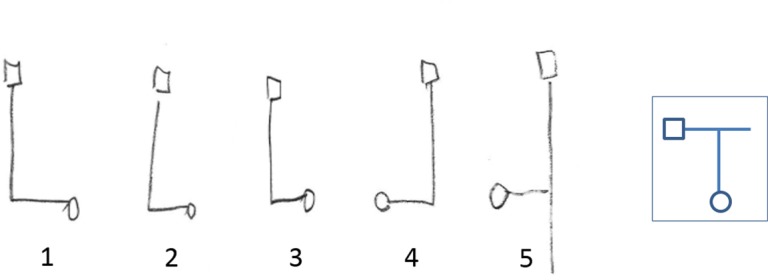
Sample drawings. Sample drawings from a senior participant, who completed maze B in the auditory, stationary condition. Showing trials 1–5 for this maze, from left to right. A circle denotes the starting point, and a square denotes the end point. Inset: a sketch of the actual maze. This example demonstrates rotation (in all 5 trials) and mirroring (in the first 3 trials, on the left) of the drawings with respect to the actual maze.

**Fig 10:**
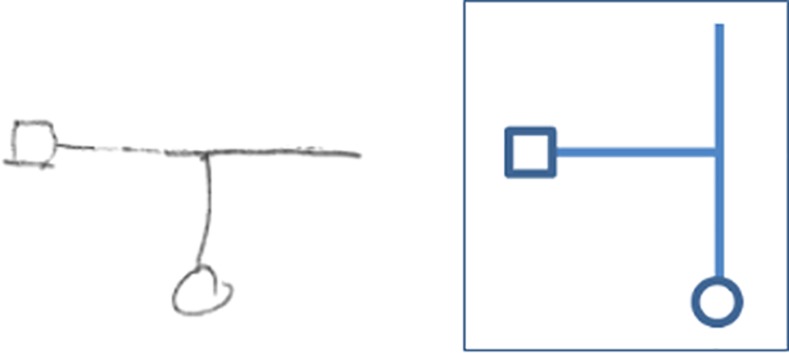
Example of a "false-corridor" drawing. On the left is a drawing made by a young participant, who completed maze D in the auditory, stationary condition. A circle denotes the starting point, and a square denotes the end point. On the right is a sketch of the actual maze. This example demonstrates what we termed a "false corridor", where a dead-end corridor was drawn at 90° to its actual direction.

The paths drawn were classified as “correct” if the maze drawn was identical to the actual maze, including cases where the participants drew the correct maze while omitting a “dead end” corridor (e.g., in the example shown in Fig 9 the last two drawings, representing trials 4 and 5, would be classified as "correct"). Classification of the mazes as “incorrect” included cases where the maze drawn was a mirror image of the correct maze (e.g., Fig 9, trials 1–3), and cases where the participants rotated a dead-end corridor (not leading to the exit) by 90° with respect to its actual direction. We termed this phenomenon “false corridor” drawing (see Fig 10).

On average, participants correctly drew about 20% of the mazes (22% in the young group, 14% in the old group, see Table 3). An additional ∼45% of the drawings represented the layout of the mazes correctly, except that they were a mirror image of the actual maze. Rotation of the drawing plane relative to the “true north” was rather common (∼70%), and was not considered incorrect (see examples of rotation in Fig 9). The pieces of paper on which the participants were asked to draw were always presented to them in a particular orientation (short edge towards the participant, long edge along the side), with the unspoken assumption that they would draw the entrance to the maze at the bottom of the page. This was not in fact the case, and we refer to the tendency to draw the starting point in a location other than the bottom of the page as a deviation from the “true north”. Overall, there were more correct mappings in the visual, compared to the auditory condition, and more in the rotating, compared to the stationary condition. Surprisingly, there were also more mirrored drawings in the visual compared to the auditory condition, more mirroring in the young vs. the old group, and more mirroring in the stationary compared to the rotating condition (see Table 3).

**Table 3:** Analysis of maze drawings. Values reported are means ± SD.

	*Visual*	*Auditory*	*Young*	*Old*	*Rotating*	*Stationary*
***% Correct mapping***	22 ± 17	16 ± 16	22 ± 9	15 ± 10	22 ± 19	15 ± 17
*% mirroring*	60 ± 25	32 ± 20	50 ± 12	40 ± 23	42 ± 21	49 ± 24

## Discussion

### Summary of main findings

The present experiment studied the effects of age, sensory modality (visual or auditory), and room rotation on movement through a virtual maze.

We found that *the sensory modality* used to navigate through the virtual maze had a significant effect on all movement parameters which we examined: time, distance travelled, number of pauses, and number of collisions with the maze walls. With the auditory cues, it took participants a longer time to complete the mazes, they took a longer path length through the maze, they paused more, and had more collisions with the walls, compared to navigation with the visual cues. *Age* had a significant effect on time, number of pauses, and number of collisions: the older group took a longer time to complete the mazes, they paused more, and had more collisions with the walls, compared to the younger group. *Rotation of the room* had no significant effect on any of the examined performance metrics. We found no significant interaction effects

### Why old are not as good as young with vision?

Our finding that the older adults took a longer time to complete the mazes is in line with previous findings showing that older adults are slower in a navigation task than younger adults [8, 24–26]. The younger group also had less pauses both when navigating with vision and with audition, and had less collisions with the wall in the visual condition, compared to the older group. The advantage shown by the younger group may be in part due to the tendency of young individuals to use an allocentric navigation strategy (relying on external cues), as opposed to an egocentric navigation strategy (relying on an internal frame of reference), preferred by older adults [24]. The younger group would thus benefit more from the visual cues available during the visual trials. It should be noted that a recent study on virtual navigation failed to find an effect of age on navigation strategy [27], and that we did not explicitly test the navigation strategy employed in the current study by the two age groups. Current models of spatial navigation suggest that both egocentric and allocentric representations contribute to spatial memory, and their relative contributions depend on the timescale of the task [28]. In this theoretical framework, on a short time scale (∼20 seconds), the egocentric representation is more pronounced, whereas on a long time scale (> 5 minutes), the allocentric representation is more active [28]. The current experimental task, which lasted approximately 3 minutes, is on the medium-term time scale, and thus would benefit from both types of representations. In addition, most of the young participants in the current study (14 out of 17) reported having regular experience with video games in their routine, compared to a minority of the older participants (3 out of 12). Potentially, that experience made it easier for the younger group to perform the task of controlling a moving avatar while being seated.

### Why the old group has a shorter path length than the young with audition?

Hearing loss is prevalent in over 60% of adults aged 70 years and older in the U.S. [29]. While traditionally, the loss of hair cells in the human ear had been considered to be the main reason for age-related hearing loss, it is now acknowledged that comprehension of auditory signals depends not only on the state of the peripheral receptors but also on the integrity of the central auditory system [30]. Deciphering the meaning of complex auditory signals, such as speech, draws on the function of brain structures outside the auditory cortex [31]. The auditory task in the current experiment required more than a mere localization of auditory cues: the participants had to maintain a mental representation of their avatar's location within the virtual environment, and continuously evaluate their distance from nearby maze walls in order to successfully negotiate the mazes. Thus, success on the auditory navigation trials depended on a combination of sensory and cognitive abilities, most likely involving the hippocampus as well as extra-hippocampal regions.

The tendency of young adults to prefer an allocentric navigation strategy, compared with an egocentric navigation strategy preferred by older adults [24] may explain the increased ability of the older group to navigate within the virtual environment in the absence of visual cues, compared with the younger group.

Rosenbaum et al [32] reported that mental navigation involves the retrosplenial cortex (directionality in an allocentric framework), the medial and posterior parietal cortex (space perception within an egocentric coordinate system) and regions of prefrontal cortex (working memory). Moffat and colleagues [33] reported that navigation through a virtual environment was associated with activation in the hippocampus, the parahippocampal gyrus, retrosplenial cortex, right and left lateral parietal cortex, medial parietal lobe and the cerebellum. Older adults performing this task showed reduced activation in the hippocampus and parahippocampal gyrus, medial parietal lobe and retrosplenial cortex, and increased activation in anterior cingulate gyrus and medial frontal lobe. Studies on the effects of aging on navigation skills have shown the older adults to have reduced abilities in some aspects of the task (e.g., took longer, and made more turning errors), but not others (e.g., recalling encountered landmarks; see [8, 25]). Importantly, results from studies on the effects of aging on navigational skills have come almost exclusively from tasks where information relied heavily on visual input (for a review, see [10]).

The current experiment is the first, to the best of our knowledge, to test the effects of aging on navigational skills using auditory cues alone, via a sensory substitution device. And so, while aging is associated with a decline in auditory function (both peripheral and central losses [30]), and a decline in several cognitive functions [5], including spatial cognition, and in it, a decline in some navigational skills [10], there are also cognitive functions that improve with age [6, 7], and which may underlie the surprising finding that the older group did better than the younger group on the auditory trials, in terms of path length. An alternative explanation to the finding that they took a shorter path through the maze, is that they employed a more conservative, less exploratory approach to navigating. This interpretation is consistent with the longer completion times found in the older group. Taken together, the older adults had an effectively reduced speed of maze negotiation, which corresponds well to findings in real-world gait measurements showing a decline in walking speed with age [34–35].

### Why was there no significant effect of rotation?

Based on previous studies [21, 22], we expected the perceptual bias that results from room rotation to interfere with task performance. However, no significant effect of room rotation was found in our current study. These findings suggest that the continuous veridical sensory feedback–whether provided visually or via the auditory sensory-substitution device–about the actual, non-biased, location of the avatar in the virtual environment, was able to overcome a perceptual bias, insofar as one resulted from the room’s rotation. Importantly, since previous studies that demonstrated the presence of a bias used much simpler sensory stimuli, it is possible that a perceptual bias was not present in the current dynamic experimental setup.

### Why navigating with auditory cues takes longer?

Though auditory cuing has been found to be more effective than visual cuing in certain situations, such as when driving in a simulator [36], spatial processing of auditory cues is done in separate neural structures than visuo-spatial information [37], and may take a different time course to process [38]. Though spatial information is shared across the senses [19], sighted individuals rely more on visual information for creating mental maps, and so the difference in performance between the two modalities may be a result of lack of sufficient training with the auditory cues.

### Why young participants had over all more correct mappings than the old group?

We found that the younger group produced an overall higher rate of correct drawings of the virtual paths they had taken. This result is consistent with findings from a study suggesting that path integration in the older population is not as good as in younger adults [39] and with findings from an experiment studying recall errors in route memorization [8]. In that experiment, the authors had young and old participants follow an experimenter through an unfamiliar route, and then recall the path. They found that the older adults made more errors, though there was no age-related difference in the "false positives" during recall (i.e., recalling a turn where there was none). It should be noted that the map drawing task relies on the translation of information derived from path integration into a map-like representation. One potential explanation is that young adults have more experience with video games, which some studies claim improve spatial cognition [40–41]. That result is consistent with the expectation that younger people, who often have experience with navigation video games, would be better able to later recall the layout of the maze they navigated through than older adults.

### Why were there so many mirror drawings?

We found that approximately half of the drawings made by the participants were mirror images of the actual paths taken. A possible explanation for this is what has been termed "mirror invariance" [42]. Presumably, humans are born with the ability to invariably recognize (and reproduce) images and their mirrored counterparts; this ability is diminished only when individuals are required to learn to read and write, where mirroring could cause confusion (e.g., for the letters p, q, b & d in the Latin script). This suppression is learned during childhood, and exists only for languages where such mirror confusion is possible, and not for others (e.g., Tamil) or for illiterate individuals [42]. Adults who have learned to repress the mirror invariance with respect to letters–in reading and writing–have retained the ability to recognize mirror images of objects, via the ventral visual pathway [42]. It may be, then, that the large portion of mirror images produced in the current experiment is the result of mirror invariance with respect to path representation.

## Conclusion

There is a decline in performance on a virtual navigation task with age and with the use of auditory feedback, as opposed to visual feedback. Nonetheless, older adults were able to successfully use a sensory substitution device to navigate through virtual mazes.

## Supporting Information

S1 Video Experimental setup. A video demonstration of navigation through the virtual mazes with visual and with auditory feedback.
(MP4)
